# Neural correlates involved in perspective-taking in early childhood

**DOI:** 10.1016/j.dcn.2024.101366

**Published:** 2024-03-19

**Authors:** M. Meyer, N. Brezack, A.L. Woodward

**Affiliations:** a Donders Institute for Brain, Cognition and Behaviour, Radboud University, the Netherlands; b WestEd, Learning & Technology, San Francisco, USA; c Department of Psychology, University of Chicago, USA

**Keywords:** Perspective-taking, Young children, EEG, Theta power, Temporal-parietal brain activity

## Abstract

Learning to consider another person’s perspective is pivotal in early social development. Still, little is known about the neural underpinnings involved in perspective-taking in early childhood. In this EEG study, we examined 4-year-old children’s brain activity during a live, social interaction that involved perspective-taking. Children were asked to pass one of two toys to another person. To decide which toy to pass, they had to consider either their partner’s perspective (perspective-taking) or visual features unrelated to their partner’s perspective (control). We analyzed power changes in midfrontal and temporal-parietal EEG channels. The results indicated that children showed higher power around 7 Hz at right temporal-parietal channels for perspective-taking compared to control trials. This power difference was positively correlated with children’s perspective-taking performance, specifically for trials in which they needed to pass the toy their partner could not see. A similar power difference at right temporal-parietal channels was seen when comparing perspective-taking trials where children’s visual access mismatched rather than matched that of their partner. No differences were detected for midfrontal channels. In sum, we identified distinct neural activity as 4-year-olds considered another person’s perspective in a live interaction; this activity converges with neural findings of adults’ social processing network.

## Highlights

• 4-year-olds’ level 1 visual perspective-taking examined during a live interaction.
• Perspective-taking modulated power (∼7 Hz) at right temporal-parietal sites.
• Power changes positively related to performance when visual access mismatched.
• Considering *differences* in viewpoints elicited a comparable neural effect.
• 4-year-olds’ neural activity was similar to adults’ social processing network.

## 1 Introduction

“Mommy, look, a dog!” “Where is it? I can’t see it from here.” Learning to account for what another person can and cannot see relative to what we can see characterizes level 1 visual perspective-taking (Flavell, 1992, Flavell et al., 1981, Moll and Tomasello, 2006). Developing perspective-taking skills is an important milestone of social cognition in early childhood. Considering another person’s perspective, and understanding that it may differ from our own, is not only pivotal for early social development, it also facilitates daily social interactions. For instance, as exemplified above, when we refer to something in the environment or attempt to coordinate our actions with another person, awareness of our social partner’s perspective allows for a more smooth and successful interaction. The development of perspective-taking skills in young children’s *behavior* has been investigated extensively; yet the neural correlates of this crucial social skill are less well understood.

### 1.1 Perspective taking in early childhood

Convergent evidence from behavioral studies indicates age-related changes in perspective-taking throughout the first years of life. For instance, Moll and Tomasello (2006) found that helping behavior of 24-month-old children, but not 18-month-olds was informed by the fact that *what* someone else sees may differ from what they themselves see (level 1 visual perspective-taking). In this study, an adult experimenter was searching for an object that she could not see, but which was visible to the child. A second object was visible for both the experimenter and the child. When the experimenter asked children for help, 24-month-olds passed the object occluded from the experimenter’s view at above-chance levels, indicating that they interpreted her behavior as searching for the object that she *could not see*. Although above chance as a group, many 24-month-olds still did not show level 1 visual perspective-taking in their behavior (Moll and Tomasello, 2006). Integrating the understanding of another person’s perspective into behavior during an interaction can prove challenging even for 3-year-old children, especially when the other person’s perspective mismatches their own (Brezack et al., 2021, Moll et al., 2013). Similarly, when engaged in a hide-and-seek game, 3-year-old children had difficulty recognizing whether someone else could see them, whereas by age four most children’s behavior indicated that they considered what another person could or could not see (Peskin and Ardino, 2003). From toddlerhood through early childhood, perspective-taking abilities progress from level 1 visual perspective-taking (understanding that *what* someone sees may differ from what you see) to more complex perspective-taking (understanding that *how* something looks to another person may differ from the way the object looks from your view; level 2 visual perspective-taking; e.g.(Moll and Meltzoff, 2011)).

### 1.2 Neural underpinnings of perspective taking

#### 1.2.1 Evidence from young children

While age-related behavioral changes in children’s perspective-taking skills have been well-studied, little is known about the neural underpinnings of perspective-taking in early childhood. To our knowledge, the only evidence about the neural underpinnings of perspective-taking early in life comes from a small set of developmental studies investigating the neural processes of Theory of Mind (i.e., understanding what another person thinks, feels, or believes). Research in adults has suggested that there is overlap in neural activity between visual perspective-taking and Theory of Mind, specifically at the left temporal-parietal junction (TPj; Theory of Mind: false belief understanding; (Schurz et al., 2013)), making research on Theory of Mind relevant for our understanding of neural underpinnings of perspective-taking. For instance, Sabbagh and colleagues (2009) measured 4-year-old children’s brain activity during a resting-state period using electroencephalography (EEG) and related this neural activity to children’s performance in Theory of Mind tasks. The results indicated that source-localized activity in the 6–9 Hz band at dorsal medial prefrontal cortex and right TPj was strongly correlated with children’s performance on the Theory of Mind tasks (Sabbagh et al., 2009). Convergent findings from Grosse Wiesmann and colleagues (2017) demonstrated that 3- to 4-year-olds’ Theory of Mind false-belief understanding was positively correlated with local white matter maturation in right TPj and medial prefrontal cortex (Grosse Wiesmann et al., 2017). Additionally, increased white matter connectivity between the inferior frontal and temporal-parietal regions was positively correlated with children’s false belief understanding. Together, these two developmental studies point to brain regions known from adult neuroimaging studies to be significantly involved in high-level social processes, namely the medial prefrontal cortex and temporal-parietal brain regions (McCleery et al., 2011, Schurz et al., 2015, Wang et al., 2016). While these high-level social processes of Theory of Mind and perspective-taking may share neural activation patterns (Schurz et al., 2013). it remains an open question which neural underpinnings specifically underlie visual perspective-taking in young children.

#### 1.2.2 Evidence from adults

Prior cognitive neuroimaging studies in adults have investigated the neural activity associated with spontaneous visual perspective-taking. For instance, in a functional magnetic resonance imaging (fMRI) study, Schurz and colleagues (2015) implemented an experiment that included trials designed to trigger spontaneous visual perspective-taking in adults, the dot task, in addition to several non-perspective-taking control trials (Schurz et al., 2015). In the perspective-taking trials, participants saw an avatar looking towards one of two walls in a room. Two dots were distributed on the walls that were either located in view of the avatar or behind the avatar (i.e., out of the avatar’s sight). Participants could always see all dots. Schurz and colleagues (2015) found that areas previously activated by high-level social processing (the right TPj, ventral medial prefrontal cortex, and ventral precuneus) showed greater activation during the visual perspective-taking trials than the control trials,. Activation in these areas was stronger when visual access between the participant and the other mismatched (i.e., on trials where the dots were in view of the participant but behind and out of sight of the avatar). McCleery and colleagues (2011) used the dot task in an EEG study with adults; findings indicated amongst others, a positive-going event-related potential around 400–500 ms over temporal parietal areas (called TP450) and a late frontal slow wave component around 700 ms (McCleery et al., 2011). Source-localization of the TP450 component suggested the right TPj as the origin, which was interpreted as an area that was important for processing another person’s perspective. However, it is a matter of ongoing debate whether the dot task actually captures visual perspective-taking (Ramsey et al., 2013, Samson et al., 2010, Schneider et al., 2017; Van Overwalle & Vandekerckhove, 2013), or whether the results may be an experimental artifact actually reflecting attentional processes (Santiesteban et al., 2014, Santiesteban et al., 2017, Vestner et al., 2022).

As such, adult neuroimaging research has also used tasks embedded in realistic, communicative settings (e.g., the director task; (Dumontheil et al., 2010)), which may assess visual perspective-taking closer to real-life. In the director task, participants are presented with several objects on a set of shelves. Participants can see all the objects on the shelves. On some trials, a “director” sits on the other side of the shelves across from the participants. Some of the shelves are *open*, which allows both the participant and the director to see the objects. Other shelves are *closed* such that only the participant (and not the director) can see the objects. In this referential communicative task, participants are asked to interact with certain objects, but they need to account for the director’s perspective to choose the correct object. Contrasting brain activity as measured by fMRI between perspective-taking (director present) versus control (director absent) trials yielded significant activity in medial prefrontal brain regions and the superior temporal sulcus (Dumontheil et al., 2010). Embedding visual perspective-taking in more realistic tasks like this communicative interaction may allow for a better understanding of the neural underpinnings of social cognition.

To investigate the oscillatory dynamics of perspective-taking in a communicative context, Bögels and colleagues (2015) made use of magnetoencephalography (MEG). In contrast with fMRI, MEG and EEG can better capture the temporal aspects of neural processing and allow for measurements of oscillatory activity. In a task designed to elicit perspective-taking, Bögels and colleagues (2015) observed modulations of theta band activity (3–7 Hz) in the right TPj and the medial prefrontal cortex, in addition to activation of language-related (temporal cortex) and memory-related (medial temporal lobe) brain areas between 350 ms – 650 ms after the critical naming onset (Bögels et al., 2015). Similarly, in an MEG and transcranial magnetic stimulation (TMS) study on mental rotation and perspective-taking, Wang and colleagues (2016) found theta power increases associated with perspective-taking localized to the right TPj (Wang et al., 2016). Seymour and colleagues (2018) replicated these findings and additionally showed that theta increases in the lateral prefrontal cortex and the anterior cingulate cortex (ACC) were also associated with perspective-taking (Seymour et al., 2018). Moreover, in their MEG study Seymour and colleagues used a Granger-causality approach to analyze the directionality of information flow with the result that frontal brain regions (lateral prefrontal cortex and ACC) were detected to exert top-down control over right TPj.

Together, findings in adults suggest that the medial prefrontal cortex and temporal-parietal brain regions are involved in visual perspective-taking, likely processed in the theta frequency range. Are these neural underpinnings of perspective-taking similar or fundamentally different earlier in life? It is possible that the temporal, spectral, or topographic distribution could differ in children; structural and functional brain development occurs throughout early childhood, particularly in prefrontal brain regions, which continue to mature until adulthood. Investigating young children’s neural processing during visual perspective-taking can help uncover how children process another person’s perspective, whether and how this may differ from similar processing in adults, and may offer insights into why children early in life struggle to integrate perspective-taking understanding into their behavior (Brezack et al., 2021, Moll et al., 2013).

### 1.3 The current study

The main question of the current, pre-registered study was: What are the neural correlates of level 1 visual perspective-taking in young children? To investigate this question, we measured 4-year-old children’s neural activity using EEG while the children were engaged in a live, communicative social interaction requiring level 1 visual perspective-taking. Four-year-old children were included in this study because they were expected to consistently pass level 1 visual perspective-taking tasks, and they were expected to be able to focus on the live interaction for multiple trials allowing for sufficient quality in the EEG signal. In the interaction, 4-year-olds were asked to pass one of two toys to a social partner. To decide which toy to pass, children had to consider either their social partner’s visual perspective (perspective-taking trials) or basic visual features unrelated to their partner’s perspective (control trials). Based on previous findings in adults, we hypothesized power changes in the theta frequency range (4–7 Hz) over midfrontal and temporal-parietal brain areas.

Specifically, we hypothesized an increase in baseline-corrected theta power over medial-frontal and temporal-parietal brain areas for perspective-taking in contrast to the control task. We additionally hypothesized a link between neural and behavioral measures of perspective-taking and a stronger increase in theta power on trials when visual perspectives on the requested toy “mismatched” (i.e., trials on which the child but not the social partner can view the requested toy: “Does Not See” trials) in contrast with trials when the visual perspectives on the requested toy “match” (i.e., trials on which both the child and the social partner can view the requested toy: “Can See” trials). For more details about our hypotheses see https://osf.io/9gbyh.

## 2 Methods

### 2.1 Participants

The preregistered goal was to include 60 children in the study (https://osf.io/9gbyh). Eleven additional children participated but were excluded due to poor signal during the study (n = 2; due to hair – signal had poor quality and could not be fixed during the impedance check), not participating in sufficient trials (n = 3 – at least 2 blocks of trials), refused to wear the EEG cap (n = 4), tech error (n = 1 – impedance was not saved and closed so no EEG data were collected during the study), or not scoring above the preregistered TPVT threshold (n = 1). This left a sample of 60 children for analysis. Within this sample (28 male, 31 female; mean age 48.2 months, range 46.1–50.8 months), children were of various descent (32 European; 7 African America, 4 Asian American, 5 Hispanic, 11 multiple races or ethnicities, and 1 did not provide information) and their caregiver with the highest education level was highly educated (41 post-graduate degree, 13 bachelor’s, 3 some college, 1 associate’s, 1 high school, 1 did not report). 25 of those children had participated in a prior behavioral study on perspective-taking one year before using a comparable set-up (Brezack et al., 2021). All details are described in the open access article (Brezack et al., 2021).

Of the sample of 60 children, 14 children did not provide sufficient valid, artifact-free trials during data analysis (see EEG data analysis for details). Thus, the final analytic sample included 46 children (22 male, 24 female; mean age 48.3 months, range 46.1–50.8 months). Caregivers reported that their children were predominantly of European descent (27; 3 Asian American, 2 African American, 2 Hispanic or Latino-American, 11 multiple races or ethnicities, 1 did not report) with high levels of parental education (highest level among both parents: 37 post-graduate, 6 bachelor’s, 2 some college, 1 did not report). 19 of those children had participated in the prior perspective-taking study one year previously. All children were exposed to English at least 75% of the time at home (because the study was conducted in English), were born within three weeks of their due dates, and had no known developmental delays. For more information on demographics, CSUS and TPVT scores for participants who were included compared to excluded in the final analysis see Supplementary Material.

The rationale to preregister the sample size in advance was 1) to allow for enough power of the estimated effect and 2) to determine a clear stopping criterion. The estimated sample size needed for a power of at least.85, with a small to medium effect size f of.2, given an alpha of.05 resulted in a minimum number of 35 participants. Note that this sample size estimation was calculated for the preregistered main analysis which was a repeated-measures ANOVA. Since we deviated from this approach in our final, post-hoc analysis (for more details see analysis sections), the power estimation is not applicable to the final analysis. While this should be considered when interpreting the current results, it is noteworthy that the final analysis contained multiple comparison correction to reduce the risk of false discovery.

Children were accompanied by their caregiver to the testing session, who provided informed consent. Children and their caregivers were informed about the testing procedure before the session, which lasted approximately one hour. The session included a perspective-taking task with a break in the middle during which a vocabulary assessment (not central to the results presented here) was administered. Before or after the session, caregivers completed two questionnaires on Qualtrics (which were also not central to the present paper): the Children’s Social Understanding Scale (CSUS; (Tahiroglu et al., 2014)) and a language questionnaire assessing children’s exposure to languages other than English. After participating in the study, families received 20 US dollars, a book or t-shirt and, if they completed the online questionnaires, a $5 Amazon gift card. This study was approved by the campus institutional review board.

### 2.2 Materials and set-up

To measure children’s perspective-taking skills on a neural and behavioral level, we adopted the same perspective-taking task as previously used with young children (Brezack et al., 2021). This live, communicative social interaction task investigates level 1 visual perspective-taking and was conducted identical to Brezack and colleagues (2021) unless stated otherwise. During this task, the child and an adult social partner (experimenter) sat across from each other at a table. On the table between the experimenter and the child was an apparatus similar to a puppet stage with two doors that could be opened by the experimenter. In front of the child were two mats, one yellow (on the child’s left) and one red mat (on the child’s right), onto which identical toys were placed during the task in view of the child. Depending on which door the experimenter opened, she could either see the toy on her left, the toy on her right, or both toys. The child could always see both toys. We used the same 16 unique toy pairs, previously used in (Brezack et al., 2021). A transparent plastic barrier prevented children from reaching for the toys and was lowered once it was the child’s turn to select a toy. As in (Brezack et al., 2021), children were asked to pass one of two toys to the experimenter. Pre-recorded verbal prompts indicated which toy to choose. To know which toy to pass, on half of the trials, children had to consider their social partner’s visual perspective (perspective-taking trials). On the other half of the trials, children had to consider basic visual features unrelated to the other’s perspective (the color of the mat on which the toy was placed; control trials). The door side that was opened (left or right) and the correct response side (left or right) were counterbalanced across trials. The perspective-taking and control trials were pseudorandomized in two orders that were counterbalanced across participants. Different from (Brezack et al., 2021), the two trial types were interleaved within each block of trials; Brezack et al. (2021) administered perspective-taking and control trials in separate blocks. The task included a familiarization phase where children saw the experimenter’s perspective, an introduction to the game, and a practice trial, followed by the perspective-taking and control trials. These trials were organized into four blocks of 8 test trials resulting in 32 trials in total with 16 perspective-taking and 16 control trials.

### 2.3 Procedure

***Familiarization:*** When children first entered the room, they were familiarized with the scene and their social partner’s perspective by walking around the table to see the view from the experimenter’s side. From here, children experienced which toy could be seen from this perspective when opening each individual door of the puppet stage set-up. Then children were accompanied back to their side of the table.

***EEG cap:*** Before beginning the perspective-taking task, the child was fitted with a 128-sensor HydroCel Geodesic Sensor Net (Electrical Geodesics, Eugene, OR, USA) which uses an online reference at electrode position Cz. Impedances were kept below 100 kΩ where possible. Note that this impedance level was relatively high. For instance, Sabbagh and colleagues (2009) reported 30 kΩ for their data collection with 4-year-old children. Still, the current impedance levels are within the bounds of the manufacturer of the HydroCel Geodesic Sensor Net (Geodesic Sensor Net Technical Manual, Electrical Geodesics, Inc.,2007). The EEG signal was digitized at 1000 Hz (Net Station software, Version 4.5.7; Electrical Geodesics). For the EEG recording, children were then seated across the table from the experimenter.

***Task setup and practice trial:*** The task was introduced as a game where children helped the experimenter collect toys for four animal friends. The animal friends shared the same view as the child; an image of each animal was placed on the clear plastic barrier separating the child from the toys such that each animal was facing towards the toys and the experimenter. The animals were introduced by pre-recorded audio prompts spoken in a child-directed voice. Pre-recording the audio prompts served two purposes, representing the animal character and controlling stimulus presentation in this live, interactive design. Children were then familiarized with the experimenter’s perspective. Two different toys (sheep and ball) were placed on the mats. The experimenter opened each door in turn and remarked on what she saw. The animal friend told the experimenter that he could see both toys, and the experimenter replied that she could only see one. Then, in a practice trial, two different toys (dog and spoon) were placed in view of the child behind the plastic barrier. The experimenter then opened both doors of the apparatus and children were prompted to pass a specific toy to their social partner (e.g., the spoon). This allowed children to gain familiarity with the procedure, which included sitting still, waiting for the plastic barrier to be lowered, and then passing a toy to the experimenter.

**Fig. 1.**
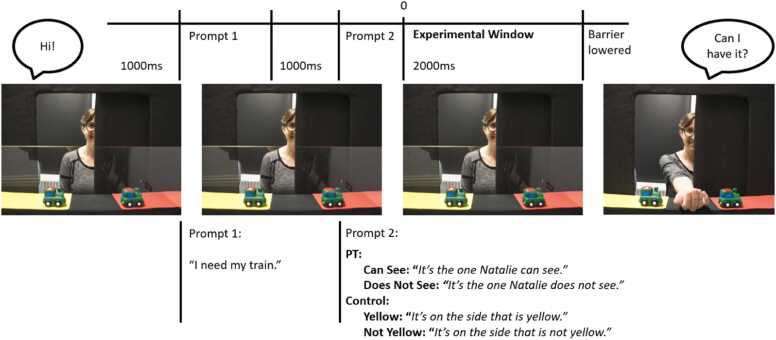
Overview of the trial structure. EEG data were time-locked to the offset of Prompt 2, i.e. the onset of the experimental time window.

***Test trials:*** The trial structure of the following test trials is illustrated in Fig. 1. Before each trial, the second experimenter placed one of two identical toys onto each mat (e.g., two small shoes) behind the plastic barrier in view of the child and the animal friend. Each trial began with the experimenter opening one of two doors of the apparatus, which allowed her to see only one of the two toys while the other was occluded from view by the closed door. The experimenter said “Hi!” followed by 1-second of silence. Then the pre-recorded voice of the animal friend stated, “I need my [*name toy*]!” (Prompt 1), followed by another 1-second delay. A second prompt (Prompt 2) specified which toy belonged to the animal: The toy either depended on the social partner’s perspective (“It’s the one that [*name experimenter*] can see!” or “It’s the one that [*name experimenter*] does not see!”; perspective-taking trials: “Can See” and “Does Not See”) or on which mat the toy was placed (“It’s on the side that is yellow!” or “It’s on the side that is not yellow!”; control trials: “Yellow” and “Not Yellow”). Note that in contrast to (Brezack et al., 2021), we used the phrase “on the side that is not yellow” instead of “on the red side” to include analogous negation in both conditions (“does not see” and “not yellow”). Following the second prompt, there was a 2-second delay before the plastic barrier was lowered and children were invited to hand one toy to their social partner, who asked, “Can I have it?” The 2-second time window preceding the child’s response served as the experimental window for the EEG analysis.

***Additional measures***: After two of the four 8-trial blocks, there was a short break for children during which the NIH Toolbox Picture Vocabulary Test (PVT) was administered. In addition to the EEG recording, the testing session was video recorded for offline analysis of children’s task performance.

### 2.4 Behavioral analysis

Children’s overt behavior was coded offline using Mangold INTERACT software to evaluate their task performance. Overt behavioral perspective-taking skills were assessed by the percentage of trials on which children correctly chose the requested toy (across all perspective-taking trials and separately for “Can See” vs. “Does Not See” trials). This analysis was also used for the control task.

In addition, on each trial, we coded whether children were turned around in their chair or verbalized, or whether caregivers interfered during the time window of interest. We also coded for instances when children explicitly said that they would disobey the task rules (e.g., “I am going to do it wrong now”) or caregivers indicated to children which toy to give the experimenter. Children’s behavioral responses on those trials were excluded.

### 2.5 EEG data analysis

To examine the neural processes involved in children’s level 1 visual perspective-taking, we focused our analysis on power changes in channels overlaying mid-frontal (E11, E16, E19, E12, E5, E4, E6 in the EGI 128-channel net) and temporal-parietal brain areas (averaged over left E30, E36, E37, E41, E42, E46, E47, E52, E53 and right E86, E87, E92, E93, E98, E102, E103, E104, E105). This pre-registered topographic focus is based on previous findings with adults (Bögels et al., 2015, Wang et al., 2016) showing an increase in theta power during a perspective-taking task and in a communicative situation requiring perspective-taking.

Net Station software was used to export the EEG data to a MATLAB-compatible format (The Mathworks, Natick, MA, USA). Data processing in MATLAB was conducted with EEGLAB (v14.0.0). Before processing the EEG data, information extracted from video coding (e.g., whether children were turned around during a trial; see Behavioral analysis for details) were imported into the EEG data as events. For pre-processing, we followed the steps of the MADE pipeline (Debnath et al., 2020). That is, after correcting for the EGI anti-aliasing time offset, the data were downsampled to 500 Hz and any discontinuous data or data recorded after the experimenter removed the EEG net at the end of the session were removed. As in (Chung et al., 2022, Debnath et al., 2020), the data were filtered between 0.3 and 50 Hz with windowed sinc FIR filters using a Hamming window (FIRfilt plugin of EEGLAB). The 24 channels on the outer layer of the 128-channel net (E17, E38, E43, E44, E48, E49, E113, E114, E119, E120, E121, E125, E126, E127, E128, E56, E63, E68, E73, E81, E88, E94, E99, E107) were removed since they mostly overlay the neck and ears. Then, artifacted channels identified by FASTER (Fully Automated Statistical Thresholding for EEG artifact Rejection; (Nolan et al., 2010)) were removed.

To correct for ocular and other muscle artifacts, independent component analysis (ICA) was performed on a copied dataset. Before running the ICA, the copied data were highpass filtered (1 Hz), segmented into 1-second epochs, and cleaned from excessive artifacts using a combined voltage and spectral threshold as in (Debnath et al., 2020) within the 20–40 Hz frequency band. In other words, filtered data (in the 20–40 Hz range) which exceeded a -/+1000 mV threshold or which fell outside the range of −100–30 dB were marked as artifacts. A channel with more than 20% artifacted epochs was removed from both the copied and original dataset. On average 3.5 out of the 102 channels were removed at this step (ranging between 1 and 6). We then used ADJUST, an EEGLAB plugin for automatized EEG artifact detection (Mognon et al., 2011) and visual inspection to identify independent components containing artifacts, which were subsequently removed from the original dataset. In our visual inspection of the components, we used two sources of information to decide whether a component was indeed indicative of an artifact. That is, we carefully inspected the components automatically identified as artifacts by ADJUST and we critically inspected the first 16 components following the guidelines for identifying components that contained blinks, saccades and muscle movements from publicly available tutorials by Mike X Cohen on this topic (https://www.youtube.com/watch?v=AKCK7DXa0gY&ab_channel=MikeXCohen). As suggested by the tutorials, components that left a doubt as to whether they reflected artifacted signal were not rejected.

As pre-registered, invalid trials were identified by children’s and caregivers’ behavior and were excluded from analysis. Trials were excluded when children verbalized (183 out of 1473 trials), children were completely turned around in their chair during the experimental window (i.e., looking away from the scene; 9 out of 1473 trials), caregivers interfered (8 out of 1473 trials), or the experimenter made an error (7 out of 1473 trials). In addition, all trials were video-coded for instances when children made an invalid behavioral response (handed both toys, neither toy, or switched sides with the toys such that their original location was no longer clear); no invalid trials were detected. After this exclusion step, children had on average 21.8 trials remaining for further analysis. Next, data were segmented to extract the 2-second experimental time window (after the critical prompt, Prompt 2, and before children’s response) with additional data for later data padding.

Following our pre-registered analyses, we initially also extracted a baseline window of 1-second preceding Prompt 2 to use for baseline-correction in the analysis. However, a growing body of literature reports that adults spontaneously process others’ perspectives (Ramsey et al., 2013, Samson et al., 2010, Schneider et al., 2017, Surtees et al., 2016); for a review see (Kampis and Southgate, 2020). Although these effects could have been driven by attentional biases based on others’ viewpoints (Santiesteban et al., 2014, Santiesteban et al., 2017, Vestner et al., 2022), it is likely that children already processed their partner’s perspective (or showed attentional biases) spontaneously once they saw the other person, even from the moment when the experimenter opened one of the apparatus doors. For this reason, this spontaneous processing before the task instruction likely contaminated the baseline period. Therefore, in our final, post-hoc analysis, instead of baseline-correcting both conditions before comparing them, we instead opted to directly compare children’s neural responses between conditions (perspective-taking versus control trials). This allowed for a comparison of moments when the task instruction was explicit, and reflects a more conservative comparison that avoids any potential bias of the baseline. For transparency, we still show figures of the pre-registered, baseline-corrected theta values in Supplementary Materials.

Next, we followed the MADE pipeline for further EEG artifact rejection. To identify any remaining ocular artifacts, a voltage threshold (±250 μV) was applied on a small set of frontal channels (E1, E8, E14, E21, E25, E32, E17), artifacted epochs were rejected in those channels, and data were interpolated in all remaining channels. Subsequently, artifacts in all remaining channels were identified and interpolated in each epoch. If an epoch contained more than 10% channels that needed to be interpolated, the epoch was rejected. On average, 3 epochs were excluded at this step. After EEG artifact rejection, twelve participants had less than 5 artifact-free trials per condition and were excluded following the pre-registered exclusion criteria.

Participants in the final sample (N = 46) contributed on average 11 trials per condition (ranging from 5 to 16 trials for perspective-taking and 6–16 trials for control trials). We did not find evidence of a relation between the number of trials included per participant and the neural effect reported below (see Supplementary Material). The remaining data were re-referenced to the average of all electrodes and then converted into current source density (CSD) using the CSD toolbox (Kayser and Tenke, 2006). Event-related spectral perturbation (ERSP) was then calculated to estimate spectral power (in dB) from 3 to 30 Hz for all channels of each trial type (perspective-taking trials: “Can See” and “Does Not See”; control trials: “Yellow” and “Not Yellow”). As pre-registered, further analysis focuses on mid-frontal (E11, E16, E19, E12, E5, E4, E6) and temporal-parietal channels (left E30, E36, E37, E41, E42, E46, E47, E52, E53 and right E86, E87, E92, E93, E98, E102, E103, E104, E105) examining power modulations in the theta frequency range (4–7 Hz; the peak in the frequency spectrum on the averaged data per participant was not clearly identifiable). Time-frequency plots of the raw power values per condition are illustrated in Supplementary Figures 4 and 5 (see Supplementary Material).

### 2.6 Final, post-hoc analysis

As mentioned above, we conducted a post-hoc analysis using a direct comparison between the two conditions to avoid any potential bias introduced by the baseline, which deviates from our pre-registered analysis. Importantly, the underlying structure of the pre-registered analysis remains analogous. That is, we investigated differential neural activity for perspective-taking (1), links between this differential neural activity and children’s behavior (2), and differential neural activity for mismatching compared to matching perspectives (3).

In particular, we first tested for differences in our main comparison, which is between perspective-taking and control conditions in the mid-frontal and temporal-parietal clusters. Instead of confining the analyses by collapsing over time and frequency, we conducted a point-wise comparison for each time and frequency point in the experimental window between 3 and 20 Hz using an non-parametric permutation test. To account for multiple comparisons, we applied false discovery rate (FDR) correction over the time x frequency point-wise comparisons per channel cluster.

Secondly, to investigate the relation between children’s neural and behavioral response, we correlated the resulting neural difference between perspective-taking and control trials with children’s performance, as was conceptually pre-registered. Note that the preregistered analysis, which compared baseline-corrected theta power between correct and incorrect trials, was not possible due to the lack of baseline (see above) and an insufficient number of artifact-free incorrect EEG trials (i.e., only about 20% of the trials were incorrect). We therefore addressed the pre-registered question about brain-behavior links by using Pearson correlations between the neural difference and children’s behavioral performance. Thirdly, to address our hypotheses on differences in perspectives, we ran the same non-parametric permutation test with FDR correction on the contrast between Does Not See trials reflecting perspectives that “mismatch” in visual access and Can See trials reflecting perspectives that “match” in visual access. As a control we ran analogous statistical comparisons between the two control trials (Yellow and Not Yellow). For all comparisons (1) and (3) the normalized difference ((A-B)/(A+B)) was compared to zero. The MATLAB analysis scripts for the final analysis are available at https://github.com/marlenemeyer/Neural-correlates-of-perspective-taking-at-age-4.git.

## 3 Results

### 3.1 Behavioral results

**Fig. 2.**
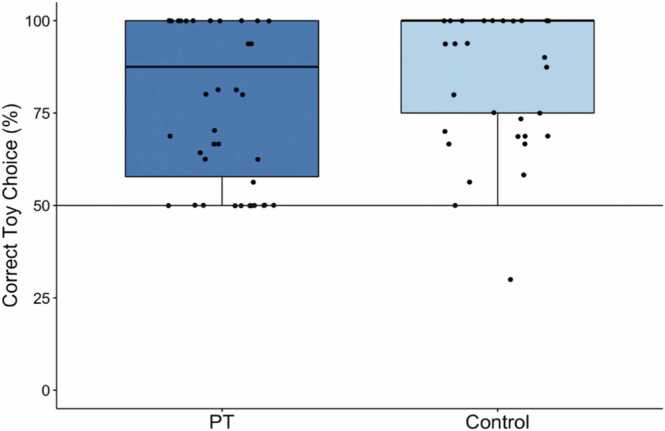
Boxplots representing the average percentage of correct toy choice for the perspective-taking (PT) and control trials. Dots represent individual participants.

Behavioral results are depicted in Fig. 2. While children performed close to ceiling level on both conditions (perspective-taking: M = 79.95, SD = 21.44; control: M = 88.40, SD = 17.41), their performance was better on the control than the perspective-taking trials (repeated-measures ANOVA: *F*(1,45) = 6.08, *p* =.018). Thus, while children considered their social partner’s perspective and translated their knowledge into action at above-chance levels (t-test to chance, 50: *t*(45) = 9.48, *p* <.001), acting on the understanding of another person’s perspective remained a non-trivial challenge. This challenge is likely affected by trials in which children’s visual access differed from that of their social partner (i.e., Does Not See trials) as is reflected in the marginally significant difference between Can See (M = 82.61, SD = 21.18) and Does Not See trials (M = 76.26, SD = 25.77) trials (paired-samples t-test: *t*(43) = 1.86, *p* =.069).

### 3.2 Results of final, post-hoc EEG analysis

**Fig. 3.**
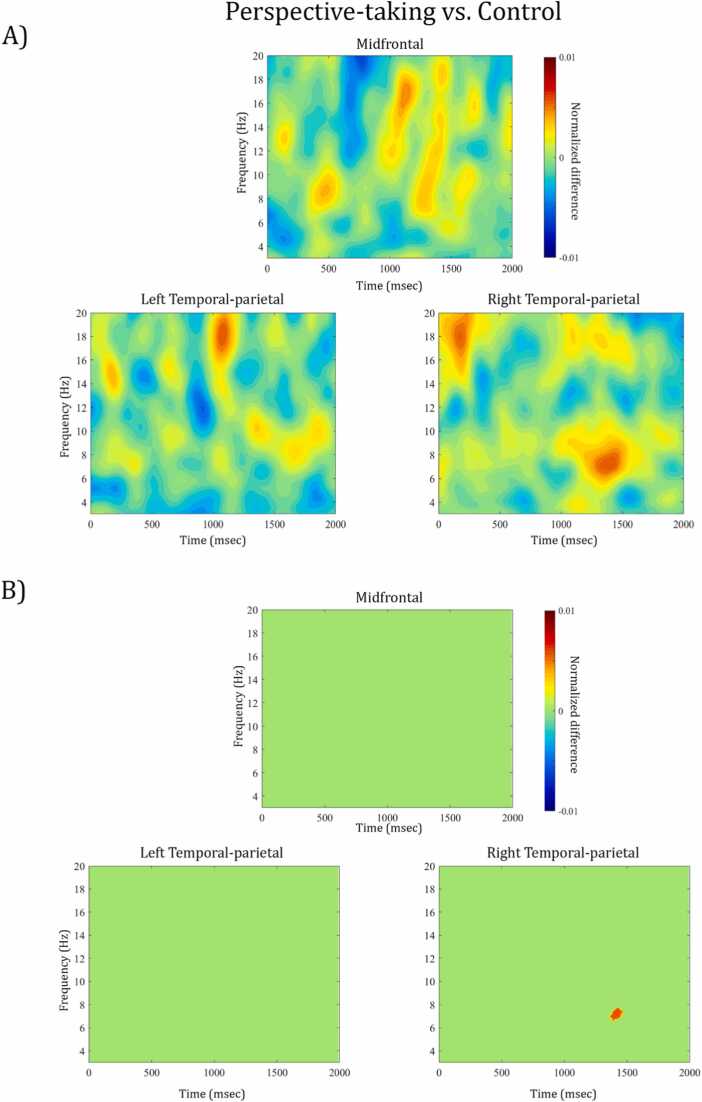
A) Time-frequency representation of the normalized difference between perspective-taking and control trials for midfrontal, left and right temporal-parietal channel clusters. Warm colors reflect an increase of power for perspective-taking compared to control trials, cooler colors reflect a power decrease, respectively. The data are time-locked to the offset of Prompt 2, i.e. the onset of the 2 second experimental window. B) Illustrates which time-frequency points show a significant power difference from zero (e.g. red area in right temporal-parietal cluster indicates significant increase in power for perspective-taking versus control) and which indicate a non-significant power difference, i.e. green areas are p >.05, FDR-corrected for multiple comparison.

***(1) Perspective-taking versus control:***Fig. 3A) displays the power differences of the direct contrast between perspective-taking ("Can See” and “Does Not See” trials) and control trials (“Yellow” and “Not Yellow”) as a function of time and frequency in the mid-frontal, left, and right temporal-parietal clusters. As visualized in the masked time-frequency plots of Fig. 3B), the non-parametric permutation test indicated a significant increase in power for the perspective-taking trials compared to the control trials at 6.2–7.7 Hz between 1368 and 1466 ms after Prompt 2 in the right temporal-parietal cluster (for time-frequency resolved information of the p- and t-values see Supplementary Figures 6 and 7). No other differences between conditions reached significance. The topographic distribution of this effect (see Fig. 6, left panel) indicated that the differential activation was specific to right temporal-parietal channels. The frequency range of this difference falls largely into the a priori defined theta band of 4–7 Hz (but see “*The role of frequency-specific activity in perspective-taking at age 4” for a more detailed discussion on frequency bands*), which is in line with our a priori hypothesis regarding the involvement of theta band activity in perspective-taking. Similarly, the direction of the observed effects, i.e., more theta power during perspective-taking than control trials, fits with our hypotheses. Moreover, the spectral and topographic features of this effect are comparable to previous adult findings of theta power increase in right temporal-parietal regions for perspective-taking (Bögels et al., 2015, Seymour et al., 2018, Wang et al., 2016).

***(2) Brain-behavior correlations:*** To investigate whether the observed neural effect was related to children’s overt performance, we computed Pearson correlations with the average neural difference between perspective-taking and control trials in relation to the average percentage of correct toy choices on 1) perspective-taking (Can See and Does Not See), 2) control (Yellow and Not Yellow), 3) Can See, 4) Does Not See, 5) Yellow, and 6) Not Yellow trials. To control for the possibility of false positives due to multiple comparisons, we used FDR-correction (with a critical value of 0.2). As a result, we found a positive correlation between the strength of the neural difference and children’s perspective-taking performance during Does Not See trials, *r(42)* =.349, *p* =.020. This small-to-medium sized effect suggests that children who showed a stronger power increase for perspective-taking compared to control trials performed better on the more demanding perspective-taking trials. None of the other correlations reached significance (all *p*’s >.05; Perspective-taking: *r(44)* =.255; Control: *r(44)* =.245; Can See: *r(44)* =.134; Yellow: *r(44)* =.276; Not Yellow: *r(43)* =.130; see Supplementary Figure 8). Thus, while we did not find evidence for an overall correlation between the neural effect and perspective-taking performance, the data support a more specific brain-behavior link, namely between the strength of the power difference between perspective-taking and control trials and children’s ability to select a toy that is visible to them but not to their interaction partner.

**Fig. 4.**
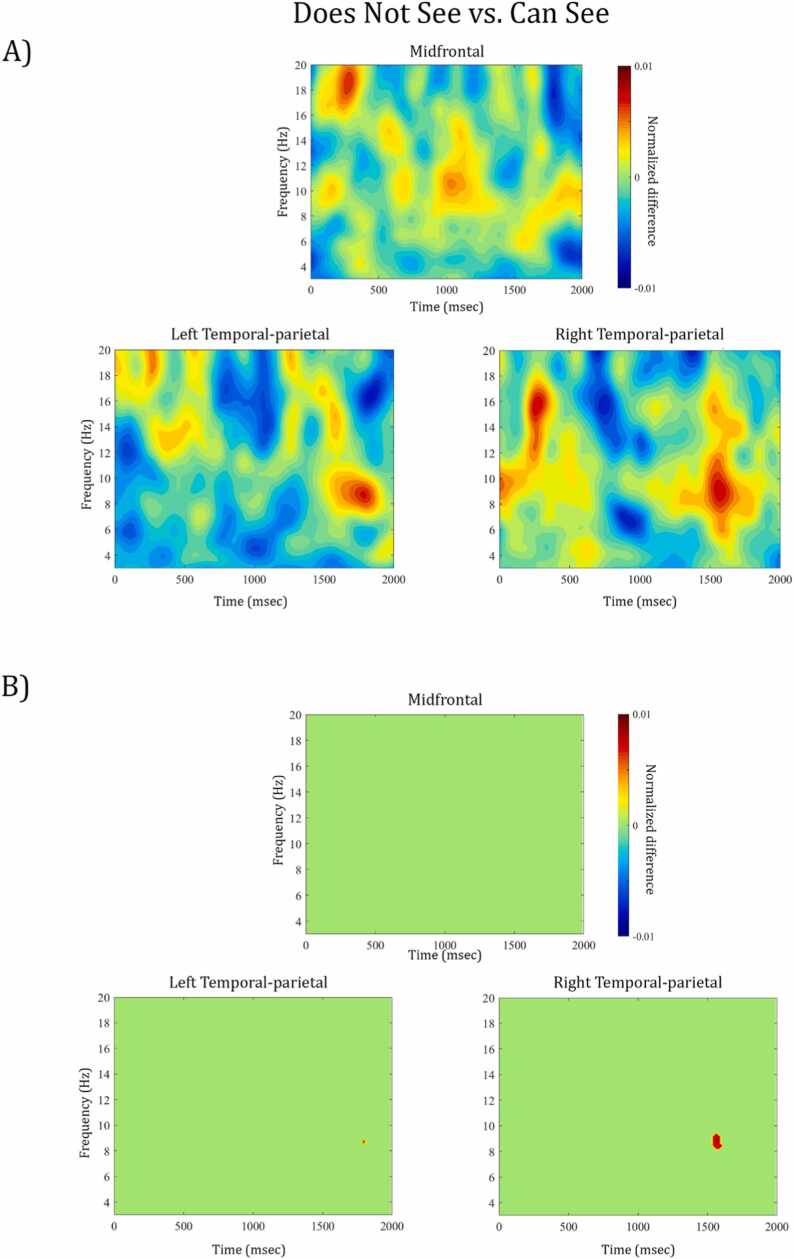
A) Time-frequency representation of the normalized difference between Does Not See and Can See trials within the perspective-taking condition for midfrontal, left and right temporal-parietal channel clusters. Warm colors reflect an increase of power for Does Not see compared to Can See trials, cooler colors reflect a power decrease, respectively. The data are time-locked to the offset of Prompt 2, i.e. the onset of the 2 second experimental window. B) Illustrates which time-frequency points show a significant power difference from zero (e.g. red area in right temporal-parietal cluster indicates significant increase in power for Does Not See versus Can See trials) and which indicate a non-significant power difference, i.e. green areas are p >.05, FDR-corrected for multiple comparison.

***(3) Differences in perspectives:*** To further examine neural activity during perspective-taking in cases when children needed to consider an object that they (but not their partner) could see, we statistically compared Does Not See with Can See perspective-taking trials. The non-parametric test indicated a significant increase in power for Does Not See compared to Can See trials at 8.2–9.3 Hz between 1544 and 1602 ms after Prompt 2 in the right temporal-parietal area (Fig. 4). This spectral distribution could reflect activity in the alpha frequency range, although the time-frequency plot (Fig. 4A) illustrates spectral leakage across multiple frequency bands (theta and alpha) potentially due to the limited number of trials available for this contrast (see more discussion on “The role of frequency-specific activity in perspective-taking at age 4”). Although significance was detected at a slightly (1–2 Hz) higher frequency band and about 200 ms later, the observed neural effect is similar to that of the main contrast described in (1) above.

**Fig. 5.**
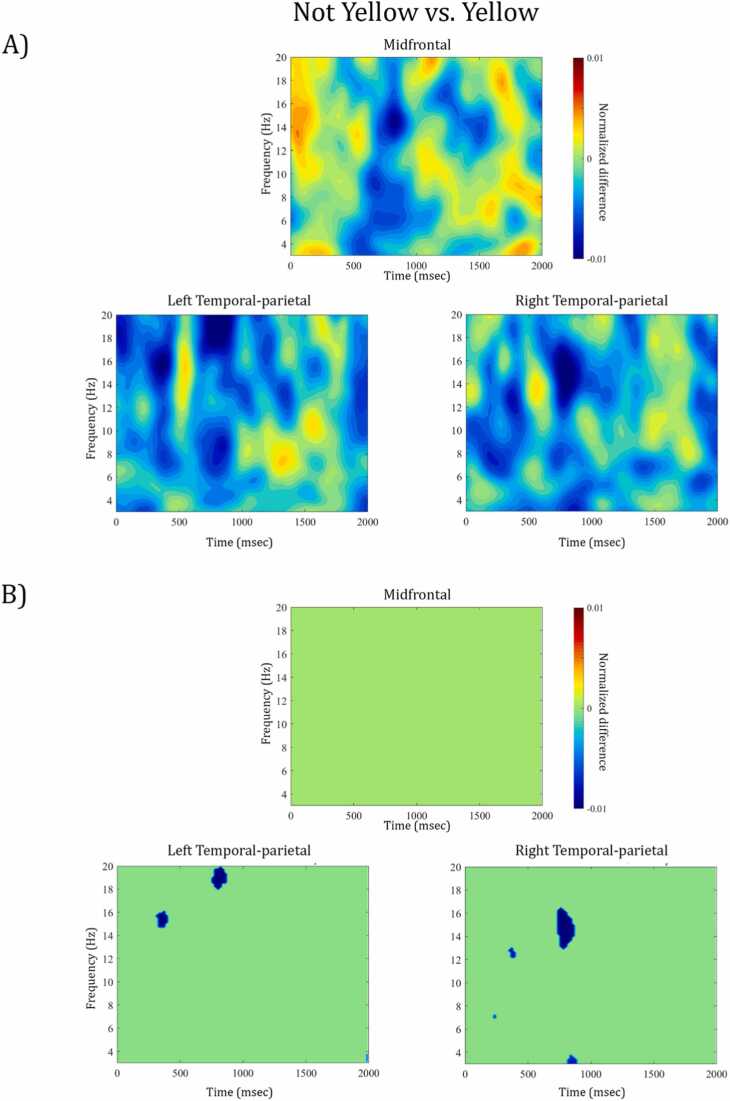
A) Time-frequency representation of the normalized difference between Not Yellow and Yellow trials within the control condition for midfrontal, left and right temporal-parietal channel clusters. Warm colors reflect an increase of power for Not Yellow compared to Yellow trials, cooler colors reflect a power decrease, respectively. The data are time-locked to the offset of Prompt 2, i.e. the onset of the 2 second experimental window. B) Illustrates which time-frequency points show a significant power difference from zero (e.g. blue area in right temporal-parietal cluster indicates significant decrease in power for Not Yellow versus Yellow) and which indicate a non-significant power difference, i.e. green areas are p >.05, FDR-corrected for multiple comparison.

**Fig. 6:**
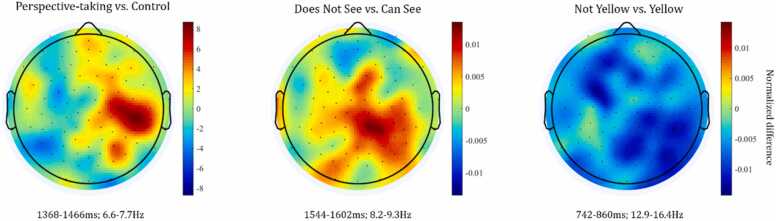
Topographic distribution of normalized difference between the perspective-taking and control trials (left), the Does Not See and Can See trials (middle), and Not Yellow and Yellow trials (right). For this topographic distribution, the time and frequency ranges which were significant for each contrast were selected and normalized difference values in these temporal and spectral ranges averaged. For the Not Yellow versus Yellow contrast, the biggest cluster in the right temporal-parietal channels was selected. Warm colors represent an increase in power for perspective-taking compared to control trials (left), an increase in power for Does Not See compared to Can See trials (middle), and an increase in power for Not Yellow compared to Yellow trials (right). Cool colors represent a decrease in power, respectively for each contrast.

We additionally contrasted Not Yellow with Yellow trials to examine whether the difference in Does Not See and Can See trials was related to the negation in the prompts rather than perspective-taking. No evidence of a significant power increase was observed in any cluster (see Fig. 5). Instead, this control contrast yielded significant power decreases in very low (∼ 3 Hz) and higher frequency bands (∼15 Hz and ∼20 Hz) in both temporal parietal clusters, a pattern distinct from both perspective-taking contrasts in (1) and (3). This suggests that the effect observed when comparing perspective types (i.e., Does Not See versus Can See) holds beyond the potential demand in resolving negation Fig. 6.

## 4 Discussion

Understanding others’ perspectives can be crucial for successful social interactions. While research with adults suggests that a network of medial prefrontal and temporal-parietal brain areas are involved in this social process, little is known about the neural underpinnings of perspective-taking during early childhood, a time of drastic changes in functional and structural brain development. In this EEG study, we set out to identify the neural correlates of level 1 visual perspective-taking in 4-year-old children. At this age, children can consider others’ perspectives during social interactions even when perspectives differ, though brain maturation is still ongoing. In this study, children engaged in a live, communicative social interaction requiring level 1 visual perspective-taking. Children passed one of two toys to a social partner either by considering their partner’s visual perspective (perspective-taking trials) or basic visual features unrelated to their partner’s perspective (control trials). While engaging in perspective-taking, the topographic andspectralfeatures of children’s neural activity was highly similar to activity previously found in studies of adults. This effect was more pronounced when children had to consider that what they saw differed from what their interaction partner could see. Further, the strength of the neural effect was related to the accuracy of children’s behavior when they a toy that they could see but their partner could not see. These findings are the first to identify neural correlates that are involved in young children’s perspective-taking.

### 4.1 Neural correlates involved in perspective-taking at age 4

Our main findings resulted from contrasting children’s neural activity during perspective-taking with activity during control trials. We found a significant increase in power between 6.2 and 7.7 Hz overlaying right temporal-parietal scalp areas approximately 1400 ms after children were prompted to consider their social partner’s perspective. Notably, children’s visual input during this time window was identical between perspective-taking and control trials; the neural effects were, by design, independent of children’s visual input. Interestingly, the topographic and spectral features of this neural effect are similar to previous findings in adults (i.e., 3–7 Hz power increase at right temporal-parietal sites during perspective-taking; Bögels et al., 2015; Seymour et al., 2018; Wang et al., 2016). We discuss potential functional implications of this spectral and topographic distribution below in more detail.

The timing of the current effect indicates that the neural differentiation follows early sensory processing (as expected within the first second). While the timing rules out an early sensory effect, we cannot be sure precisely which processes may be involved in the effect. It remains an open question whether the neural effect captures the processing of another person’s visual perspective, the decision-making process children engage in to choose a toy, or the integration of this information into their action plan after choosing a toy. Still, the neural effect likely captures a crucial aspect of perspective-taking, namely processing that what children can see differs from what someone else can see; this is evident in the main contrast, the contrast between Does Not See and Can See trials, and the brain-behavior link.

Also, while some theories of perspective-taking dichotomize separate rapid and slow processes (Ramsey et al., 2013, Samson et al., 2010; Van Overwalle and Vandekerckhove, 2013), others suggest that the timing of processing another person’s perspective is instead driven by contextual factors (Dale et al., 2018). For instance, the need for a fast response or salient perceptual features may lead to faster processing than contexts that allow for more processing time or are more difficult. In this social interaction task, two seconds separated the prompt to consider another’s perspective and the moment when children could respond (i.e., the barrier was lowered for children to pass a toy to their partner). The observed neural response emerged towards the end of the two-second time window, which may indicate that there were high task demands, such as needing to consider the other person’s viewpoint and planning a response. It remains an open question whether the precise timing of this neural effect is generalizable to other perspective-taking contexts.

***Link to behavior:*** We found a positive relation between the neural effect and children’s perspective-taking performance on trials in which children needed to pass the toy that their partner could not see. In other words, children who showed a greater difference in neural activity for perspective-taking than control trials were better able to integrate another person’s perspective into their behavior when children’s perspective differed from that of their partner. We found no evidence for a general relation with perspective-taking performance, nor with children’s performance on the control trials. While the absence of evidence should be interpreted with caution, this pattern of evidence may point to a specific link between the identified neural activity with processing differing viewpoints, rather than with processing others’ perspectives in general or with more domain-general processes. Together, these findings provide the first indication that the observed neural effect is linked to children’s processing of others’ differing viewpoints. Several questions arise from these findings: Does the strength of this neural effect predict children’s perspective-taking abilities later in life? When does this neural activity pattern emerge? Is this neural correlate visible in infancy when infants’ behavior does not yet indicate perspective-taking understanding? The identification of this neural effect allows these questions to be addressed empirically in future research.

***Differences in perspectives:*** In addition to the main contrast of interest, we also examined whether children’s neural activity differed by tasks with different perspective-taking demands. In particular, we contrasted neural activity between trials when children considered overlapping visual access (i.e., toys that the child and their partner could see) and with trials featuring mismatched visual access (i.e., toys that the child, but not their partner, could see). Interestingly, though significant at a slightly higher (1–2 Hz higher) frequency and about 200 ms later, the observed neural effect of this contrast closely resembled the neural activity identified by the main analysis. In addition, the power increase was also observed over right temporal-parietal scalp areas. Specifically, greater power was observed when children considered how their viewpoint differed from that of their interaction partner than when children considered shared visual access to an object. The similarities with the main contrast and the link with behavior suggest that the current findings could reflect a more specific, yet pivotal aspect of perspective-taking, namely processing the difference between children’s own and another’s visual access. Moreover, these findings align with recent neuroimaging evidence in adults (Kolling et al., 2021). When adults considered visually presented information that was occluded from someone else’s view, they showed increased activation of brain areas around the right TPj (Kolling et al., 2021). Kolling and colleagues (2021) suggested that temporal-parietal brain areas may be particularly sensitive to information that is different for oneself compared to others. This notion is coherent with a large body of converging evidence of adult neuroimaging research for the involvement of TPj in social processing such as self-other distinction and conflicting perspectives (e.g., Kampis and Southgate, 2020; Mars et al., 2012; Steinbeis, 2016; see below). It is also in line with recent suggestions of a unifying account of right TPj function proposing that activity in this area reflects the processing of mismatch, in this case between perspectives (Doricchi et al., 2022). Since TPj has also been associated with attentional processes (Decety and Lamm, 2007), it is possible that task difficulty, which could increase attentional demands, may be driving this neural effect. However, no increase in power was observed in temporal-parietal or medial prefrontal scalp areas when comparing a more attentionally-demanding task (Not Yellow; “It’s on the side that is not yellow!”) with a less demanding task (Yellow; “It’s on the side that is yellow!”). Task demand alone is therefore unlikely to explain the current findings. Along the same lines, attentional orienting and re-orienting have been associated with activity in different subregions of TPj in adults (Dugué et al., 2018). It is possible that in the perspective-taking condition, when the name of the interaction partner was mentioned, children might have re-oriented their visual attention away from the toys towards the person. This would have been less likely in the control condition, potentially yielding an alternative explanation for these findings. However, the visual match and mismatch conditions within the perspective-taking trials do not contain this confound. The neural activity pattern seen when comparing the match and mismatch conditions, together with the observed link with children’s performance on mismatch trials, suggests that the identified neural activity is involved in processing differences between another person’s perspective and one’s own, rather than solely reflecting attentional re-orienting.

### 4.2 The brain areas involved in perspective-taking at age 4

The current findings identified differential activity at right temporal-parietal electrode sites as children processed another person’s perspective. No evidence was found for the involvement of medial prefrontal cortex in this study. Given the low spatial resolution of EEG and the lack of localization of the source of this activity, it remains an open question where this neural activity originated. Still, children’s differential neural activation at right temporal-parietal electrode sites converges with previous evidence from cognitive neuroscience research with adults demonstrating the involvement of right temporal-parietal brain regions in perspective-taking (Bögels et al., 2015, Kolling et al., 2021, Wang et al., 2016). For instance, several MEG studies detected increased 3–7 Hz theta power in right TPj for situations that require perspective-taking (Bögels et al., 2015, Seymour et al., 2018, Wang et al., 2016). Similarly, results from fMRI research and studies using transcranial direct current stimulation (tDCS) and transcranial magnetic stimulation (TMS) showed activation of comparable brain areas (Kolling et al., 2021, Santiesteban et al., 2012, van Elk et al., 2017). Together, these studies provide causal evidence for the importance of right TPj in processing the difference between another person’s viewpoint and one’s own (for an extended discussion see Supplementary Material).

A recent proposal by Kolling and colleagues (2021) based on fMRI findings in adults predicts that a larger network, including the medial prefrontal cortices (ventral medial prefrontal cortex; vmPFc and dorsal medial prefrontal cortex; dmPFC), is involved in processing information about others, Kolling and colleagues postulated a two-step process: The first step entails extracting information available to oneself (egocentric knowledge). The second step is putting this knowledge into context and updating it depending on what is known about the other person’s view and their access to information (Kolling et al., 2021). While medial prefrontal cortices are involved in extracting (vmPFC) and modifying (dmPFC) egocentric information, TPj was proposed to process how the other person’s perspective *differs* from one’s own and subsequently feeds this information to frontal brain regions. This is particularly interesting in relation to the social interaction in the present study. The egocentric information was kept constant between the perspective-taking and control trials (i.e., children received the same visual input in all conditions). At the same time, we manipulated the importance of considering the other’s visual perspective, which either matched or mismatched that of the child. This may explain the differential neural response at right temporal-parietal electrode sites without a differential response at medial prefrontal electrode sites. Interestingly, recent evidence from tDCS research with adults similarly highlights the role of right temporal-parietal but not dorsomedial prefrontal cortex in perspective-taking (Martin et al., 2020).

Alternatively, while interpreting the absence of evidence for midfrontal activation with caution, it is possible that frontal cortices become increasingly involved in perspective-taking throughout early childhood; thus, perspective-taking processes may still be in development. In fact, prefrontal brain areas are the latest to mature in childhood, and building functional connections in a network with temporal-parietal brain areas might still be ongoing. Comparisons between children of different ages may provide insights on this matter in future research.

### 4.3 The role of frequency-specific activity in perspective-taking at age 4

What are the implications of the spectral distribution of the current neural findings? The main neural effect was identified at around 7 Hz. This is in line with activity in the 3–7 Hz theta frequency range previously found in right temporo-parietal areas during perspective-taking in adults (Bögels et al., 2015, Seymour et al., 2018, Wang et al., 2016) and was thus consistent with our a priori hypothesis on the involvement of activity in the 4–7 Hz theta range in perspective-taking early in life. Besides this, the spectral distribution is also consistent with findings of Sabbagh and colleagues (2009) who showed source-localized activity in the 6–9 Hz band at right TPj to be correlated with children’s Theory of Mind performance. Sabbagh and colleagues interpreted activity in the 6–9 Hz band in their study as alpha band activity. EEG characteristics, like theta and alpha peak frequency, undergo drastic changes in early childhood (Cellier et al., 2021, Marshall et al., 2002, Orekhova et al., 2006, Thorpe et al., 2016) and categorization of frequencies into bands is likely dependent on the underlying functional processes and the topographic source of the respective activity. Together, this makes identification and dissociation of specific frequency bands like theta and alpha particularly difficult. In our study, we defined the frequency band of interest (4–7 Hz), which we interpreted as theta based on previous research on children (Orekhova, Stroganova, Posikera, and Elam, 2006) and on perspective-taking research with adults (Bögels et al., 2015, Wang et al., 2016), a priori. It remains an ongoing debate in developmental cognitive neuroscience whether theta oscillations play a functional role and the extent to which this role may differ dependent on their source. Active learning, top-down processes like attention and memory, conflict monitoring, and processing prediction errors have been associated with changes in theta power (Begus and Bonawitz, 2020, Buzzell et al., 2019, Köster et al., 2021, Meyer et al., 2019, Meyer et al., 2023, van der Velde et al., 2021). Across infancy, the topographic distribution of theta networks shifts from parietal-occipital to frontal-parietal networks and theta networks, in contrast to alpha, selectively gain connectivity strength for social stimuli (van der Velde et al., 2021). While this might suggest a particular role for theta band activity in social processing, others propose that theta power reflects more fundamental processing features such as processing prediction errors (Köster et al., 2021). Minimizing prediction errors improves internal models and predictions about the (social) world. In perspective-taking, the viewpoint of the self and the interaction partner are typically different, which could lead to a conflict of perspectives potentially processed as prediction error. This would also be consistent with the current findings showing a power increase for a larger mismatch in visual access (i.e., larger prediction error). Similarly, adult findings reported larger theta power increases with larger differences between one’s own and another’s viewpoint (Seymour et al., 2018). Considering modulations in theta band activity in the light of fundamental information processing principles like generating predictions and prediction errors may be a fruitful avenue for future research to better understand higher social cognition. In this context, the use of computational modeling is particularly promising as it allows for formalizing and testing the precise processes involved.

The current findings could also be linked alpha band activity. As discussed by Jones and colleagues (2015), in addition to theta, alpha band activity was found to be sensitive early in life to processes involving other people. For instance, a large body of literature associated changes in central alpha power, also called mu rhythm, with the processing of other people’s actions (e.g., Fox et al., 2016; Stapel et al., 2010; Marshall and Meltzoff, 2011). In the current analyses of matched versus mismatched visual access, power changes reached significance at 8.2–9.3 Hz, which could reflect activity in the alpha frequency range. However, descriptively, the time-frequency plot of this contrast (Fig. 4A) shows spectral leakage across multiple frequency bands (theta and alpha). Therefore, interpretations about the spectral specificity of this contrast should be considered with caution.

While the current findings are consistent with the a priori defined theta frequency range and adult neural correlates of perspective-taking in the theta band, they also link to literature on alpha band activity. Therefore, it remains important to consider the specific frequencies identified here. Using information on the specific frequencies involved in the current neural effect will also aid in generating hypotheses for future studies irrespective of categorizing into pre-defined frequency bands.

## 5 Conclusion

In sum, we identified distinct neural activity as 4-year-olds considered another person’s perspective in a live, social interaction. These findings offer the first evidence of neural correlates that reflect key aspects of perspective-taking in early childhood. In particular, this neural activity appears to be related to processing the difference between one’s own and another person’s viewpoint. Moreover, the neural effect identified in 4-year-olds converges with findings on the role of the right temporo-parietal cortex in adults’ social processing network. This distinct neural activity associated with key elements of visual perspective-taking early in life may serve future investigations on the emergence of perspective-taking skills and related underlying neural changes.

## Funding information

This work was supported in part by the NIHCD grant under [Grant P01 HD064653] at the University of Chicago.

## CRediT authorship contribution statement

MM, NB and AW jointly developed the study concept and design. NB and MM collected the data. MM and NB performed data analyses and all authors interpreted the data. MM drafted the manuscript, and NB and AW contributed critical revisions. AW acquired financial support for the project leading to this publication. All authors approved the final version of the manuscript for submission.

## Declaration of Competing Interest

The authors declare that they have no known competing financial interests or personal relationships that could have appeared to influence the work reported in this paper.

## Data Availability

The EEG data in BIDS format and MATLAB analysis scripts using the open-source EEGLAB toolbox are available on the Open Science Framework https://osf.io/au5z7/ (DOI 10.17605/OSF.IO/AU5Z7).
