# Supplementary material for: Neural correlates involved in perspective-taking in early childhood
Source: Dev Cogn Neurosci. 2024 Mar 19;66:101366. doi: 10.1016/j.dcn.2024.101366 (PMC10965458; doi:10.1016/j.dcn.2024.101366)
Supplement: Supplementary file 1 — Supplementary material. [file mmc1.pdf]

Supplementary Materials:

Neural correlates involved in perspective-taking in early childhood

Preregistered baseline-corrected analyses.

In the pre-registration of this study (https://osf.io/9gbyh), we proposed analyses based on baseline-corrected data. However, a growing body of literature reports spontaneous processing of others' perspectives (Samson et al., 2010; Surtees, Samson & Apperly, 2016; for a review see Kampis & Southgate, 2020). Although these effects could also have been driven by attentional biases based on others' viewpoints (Santiesteban et al., 2017; Vestner et al., 2022), it is likely that children already processed their partner's perspective (or showed attentional biases) spontaneously once they saw the other person, even from the moment when the experimenter opened one of the apparatus doors. For this reason, this spontaneous processing before the task instruction likely contaminated the baseline period. Therefore, in our final, post-hoc analysis, instead of baseline-correcting both conditions before comparing them, we instead opted to directly compare children's neural responses between conditions (perspective-taking versus control trials). This allowed for a comparison of moments when the task instruction was explicit, and reflects a more conservative comparison that avoids any potential bias of the baseline. The results of the post-hoc analysis are described in the main manuscript. For transparency, we present outcomes of the pre-registered, baseline-corrected theta power analyses in which the baseline window of 1-second preceding Prompt 2 was log subtracted from the window of interest (i.e., log(window-of-interest)-log(baseline)). All trials and participants used in the final, post-hoc analysis were also used in this baseline-corrected analysis. EEG data are publicly available under https://osf.io/au5z7/ (DOI 10.17605/OSF.IO/AU5Z7).

Perspective-taking vs. Control. When testing for differences in baseline-corrected theta power in a within-subjects ANOVA with Condition (PT, Control) and Area (midfrontal, left temporal-parietal, right temporal-parietal), neither of the main effects (Condition: $F(1, 38) = .124$, $p = .727$. partial eta squared = .003; Area: $F(1.7, 64.637) = 1.043$, $p = .348$. partial eta squared = .027) nor the interaction effect (Condition * Area: $F(2, 76) = 1.199$, $p = .307$, partial eta squared = .031) showed significant differences. Note that Mauchly's test indicated that the assumption of sphericity had been violated for Area, $\chi^2(5) = 7.154$, $p = .028$, therefore degrees of freedom were corrected using Greenhouse-Geisser estimates of sphericity ($\varepsilon = .85$).

As pre-registered, we also ran one-samples t-tests for midfrontal and temporal parietal areas which resulted in a significant difference from baseline for all areas in the perspective-taking condition (midfrontal: $t(41) = -2.391$; $p = .021$, Cohen's $d = -.369$; left temporal-parietal: $t(39) = -3.681$; $p < .001$, Cohen's $d = -.582$; right temporal-parietal: $t(40) = -5.392$; $p < .001$, Cohen's $d = -.582$) and the control condition (midfrontal: $t(42) = -2.980$; $p = .005$, Cohen's $d = -.455$; left temporal-parietal: $t(41) = -4.818$; $p < .001$, Cohen's $d = -.743$; right temporal-parietal: $t(40) = -3.625$; $p < .001$, Cohen's $d = -.566$) such that power was decreased with respect to the baseline window. Because the baseline precedes the experimental time window, children can see the other person and process their viewpoint during this time. Although speculative, these results may suggest spontaneous processing of another person's perspective (Samson et al., 2010; Surtees, Samson & Apperly, 2016; for a review see Kampis & Southgate, 2020) or attentional processing based on another person's viewpoint (Santiesteban et al., 2017; Vestner et al., 2022) during baseline.

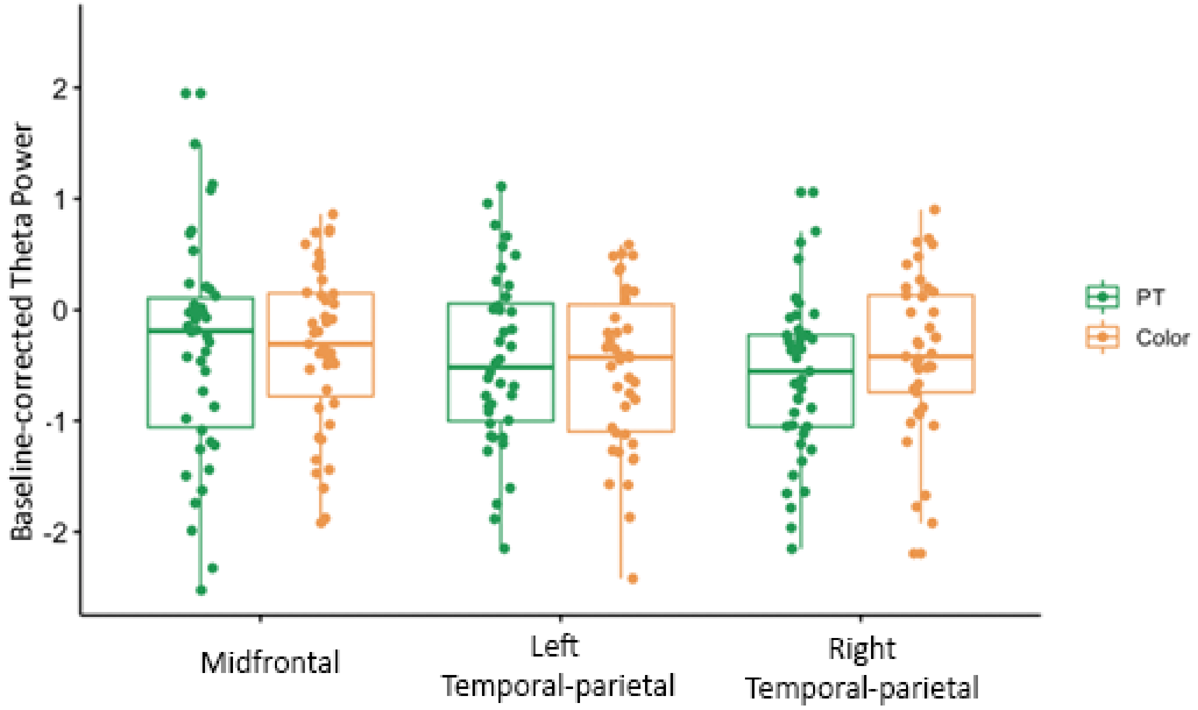

Supplementary Figure 1. Baseline-corrected power over the 4-7Hz frequency range in the midfrontal, left and right temporal-parietal channel clusters separated per condition (PT: perspective-taking and Color: Control). Dots represent individual participants.

We also pre-registered comparing phase coherence between the perspective-taking and control condition. For this purpose, we extracted the weighted phase-lag index between midfrontal and left/right temporal-parietal clusters. Weighted phase-lag values were compared in a within-subjects ANOVA with Condition (PT, Control) and ChannelPair (midfrontal-left temporal-parietal, midfrontal right temporal-parietal). The main effect of Condition did not reach significance (Condition: $F(1, 42) = .026$, $p = .874$. partial eta squared = .001). The main effect of ChannelPair was significant (ChannelPair: $F(1, 42) = 4.104$, $p = .049$, partial eta squared = .089), showing generally higher connectivity between midfrontal and right temporal-parietal (Mean = .332; STD = .059) as opposed to left temporal-parietal clusters (Mean = .319; STD = .056). The interaction effect did not reach significance (Condition * ChannelPair: $F(1, 42) = .844$, $p = .363$, partial eta squared = .02).

Brain-Behavior. When testing for differences in baseline-corrected theta power preceding a correct or incorrect behavioral response in a within-subjects ANOVA with Condition (Correct, Incorrect) and Area (midfrontal, left temporal-parietal, right temporal-parietal), neither of the main effects (Condition: F(1, 22) = 2.317, p = .142. partial eta squared = .095; Area: F(2, 44) = 1.530, p = .228, partial eta squared = .065) nor the interaction effect (Condition * Area: F(2, 44) = 1.114, p = .337, partial eta squared = .048) showed significant differences (Supplementary Figure 2). Note that this only contains a subset of participants (*N = 23*), namely those with sufficient artifact-free correct *and* incorrect trials in the perspective-taking condition.

In addition, as pre-registered, we conducted Pearson correlations with implicit PT performance (proportion looking to the target toy) and baseline-corrected theta power during PT trials for each area. None of the correlations reached significance (midfrontal: *r*(38) = -.169; *p* = .298; left temporal-parietal: *r*(37) = .203; *p* = .208; right temporal-parietal: *r*(38) = .217; *p* = .178). For this analysis, all participants with sufficient artifact-free EEG data and usable behavioral data were used.

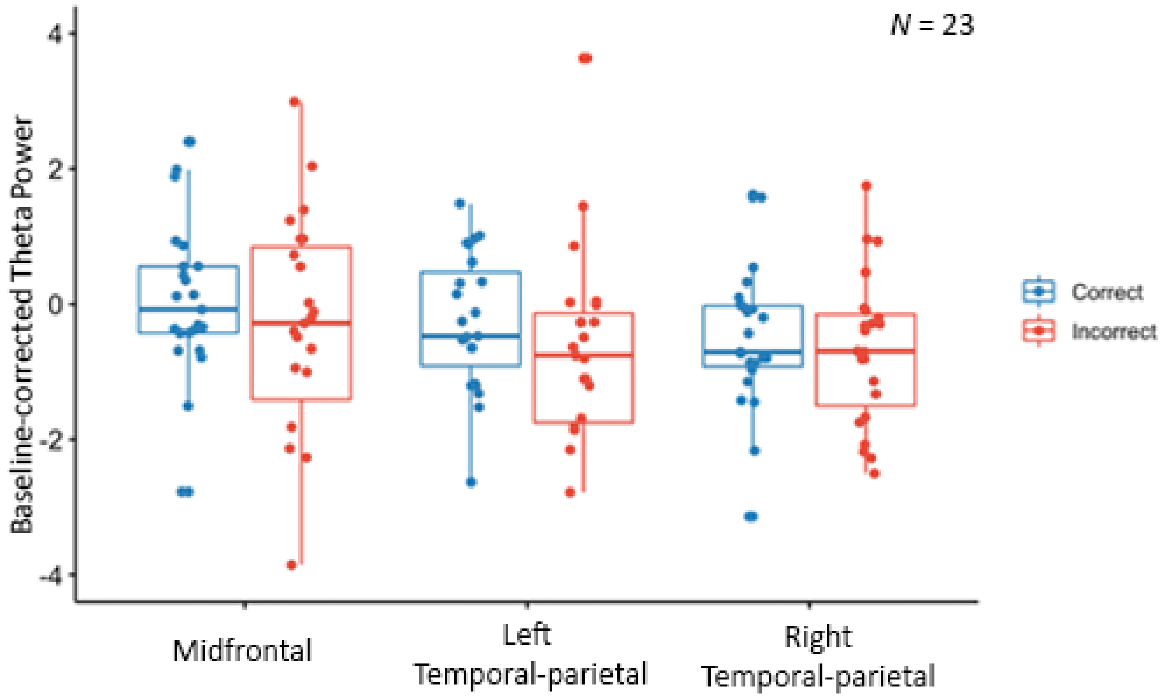

Supplementary Figure 2. Baseline-corrected power over the 4-7Hz frequency range in the midfrontal, left and right temporal-parietal channel clusters separated for whether the neural activity preceded a correct and incorrect response during perspective-taking trials only. Dots represent individual participants.

Difference in perspectives. When testing for differences in baseline-corrected theta power in a within-subjects ANOVA with Condition (Does Not See, Can See) and Area (midfrontal, left temporal-parietal, right temporal-parietal), neither of the main effects (Condition: $F(1, 39) = 1.123$, $p = .296$. partial eta squared = .028; Area: $F(2, 78) = .830$, $p = .440$, partial eta squared = .021) nor the interaction effect (Condition * Area: $F(2, 78) = .373$, $p = .690$, partial eta squared = .009) showed significant differences.

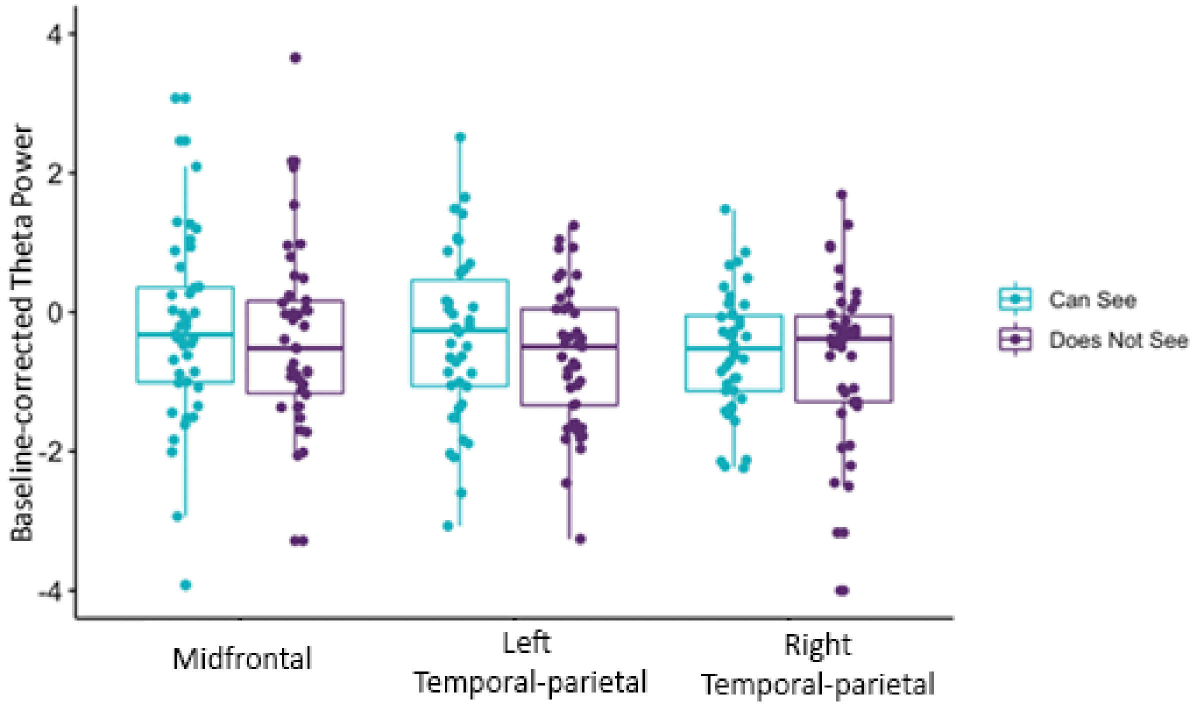

Supplementary Figure 3. Baseline-corrected power over the 4-7Hz frequency range in the midfrontal, left and right temporal-parietal channel clusters separated by different perspective-taking trial types (i.e., Can See and Does Not See trials). Dots represent individual participants.

Further details on the final, post-hoc analysis

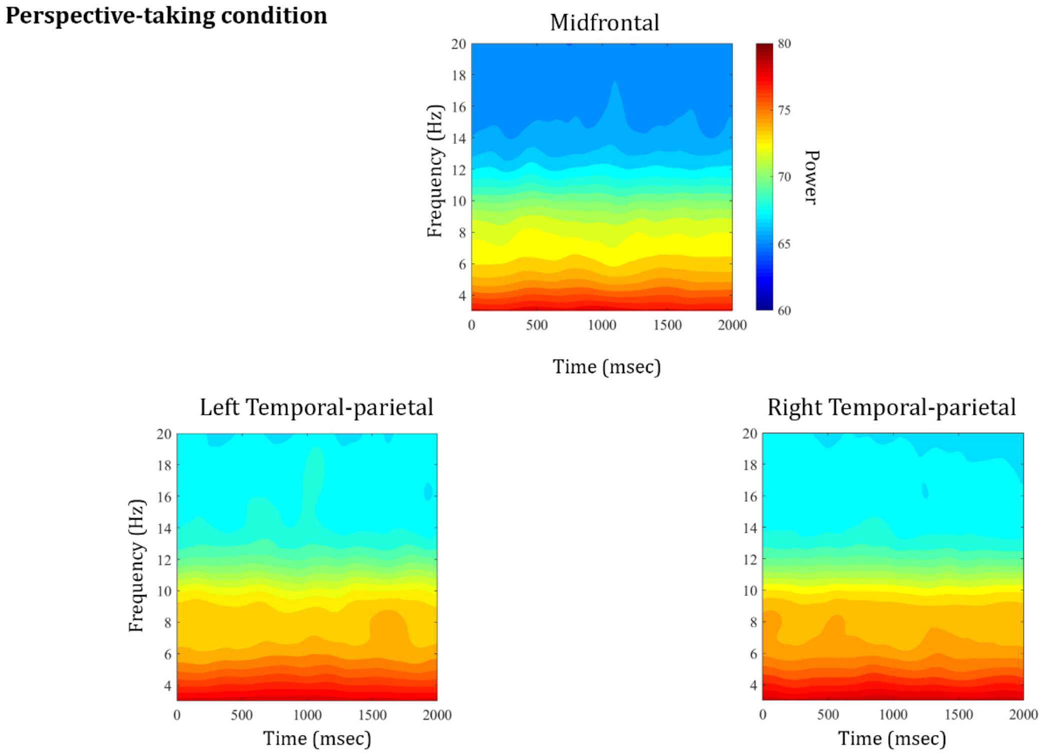

Supplementary Figure 4. Time-frequency representation of the raw power values for the perspective-taking condition for midfrontal, left, and right temporal-parietal channel clusters. The data are time-locked to the offset of Prompt 2, i.e., the onset of the 2 second experimental window.

Control condition

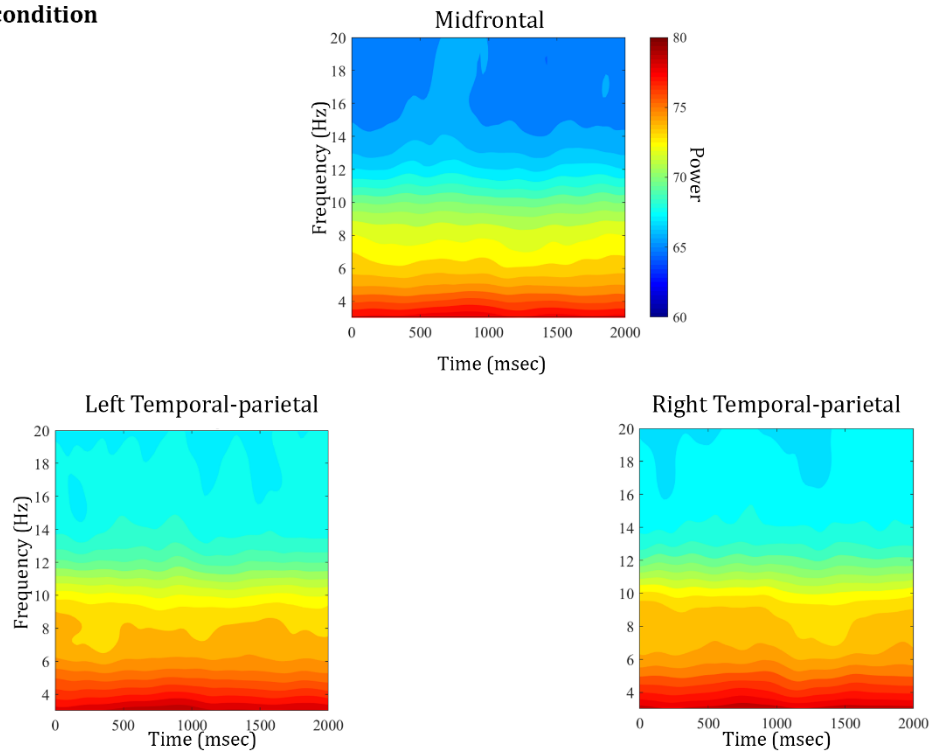

Supplementary Figure 5. Time-frequency representation of the raw power values for the control condition for midfrontal, left, and right temporal-parietal channel clusters. The data are time-locked to the offset of Prompt 2, i.e., the onset of the 2 second experimental window.

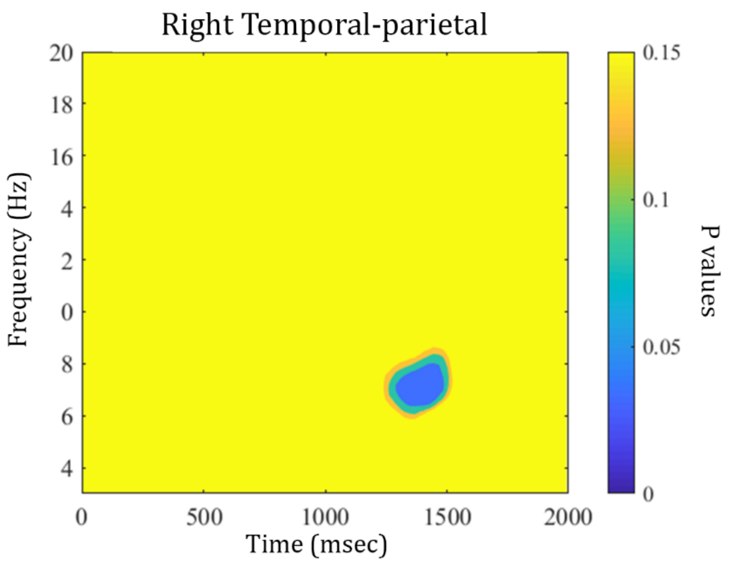

Supplementary Figure 6. Time-frequency representation of the p-values for the main contrast between perspective-taking and control trials for the right temporal-parietal channel cluster. Warm colors reflect higher p-values, cooler colors reflect lower p-values, respectively. The data are time-locked to the offset of Prompt 2, i.e., the onset of the 2 second experimental window.

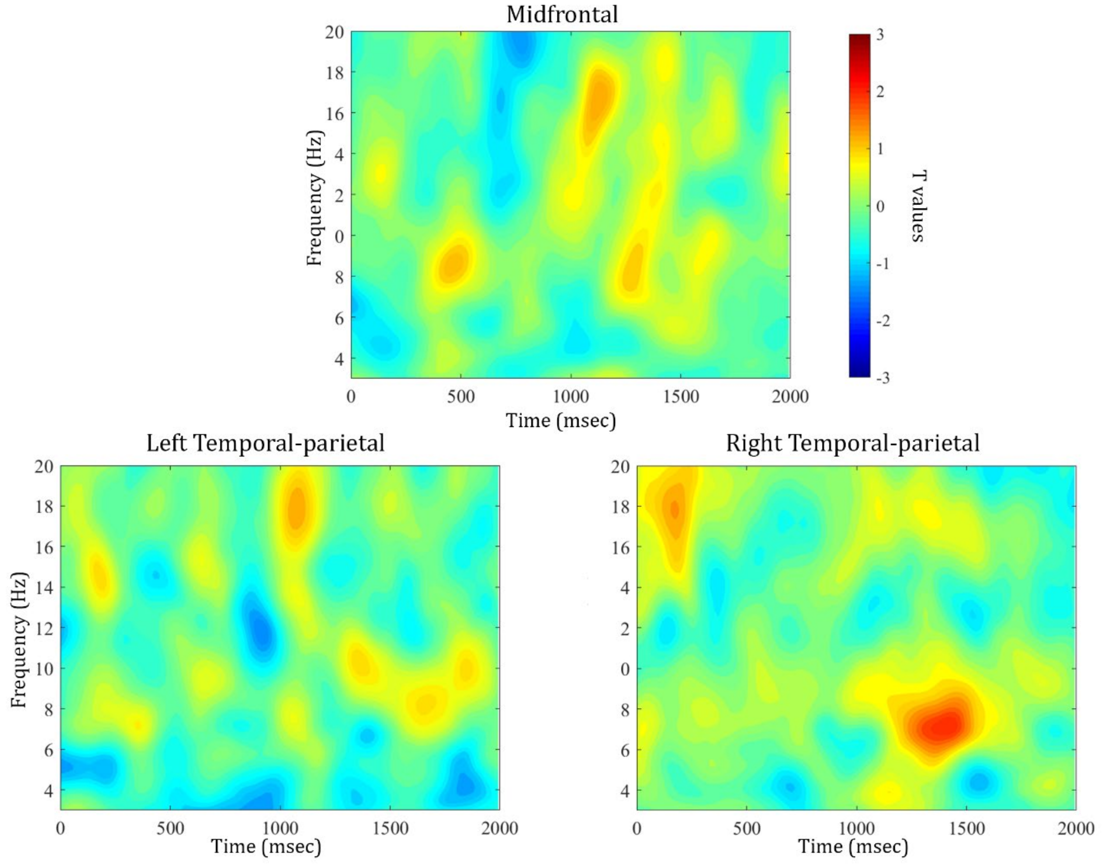

Supplementary Figure 7. Time-frequency representation of the t-values for the main contrast between perspective-taking and control trials for midfrontal, left and right temporal-parietal channel clusters. Warm colors reflect higher t-values, cooler colors reflect lower t-values, respectively. The data are time-locked to the offset of Prompt 2, i.e., the onset of the 2 second experimental window.

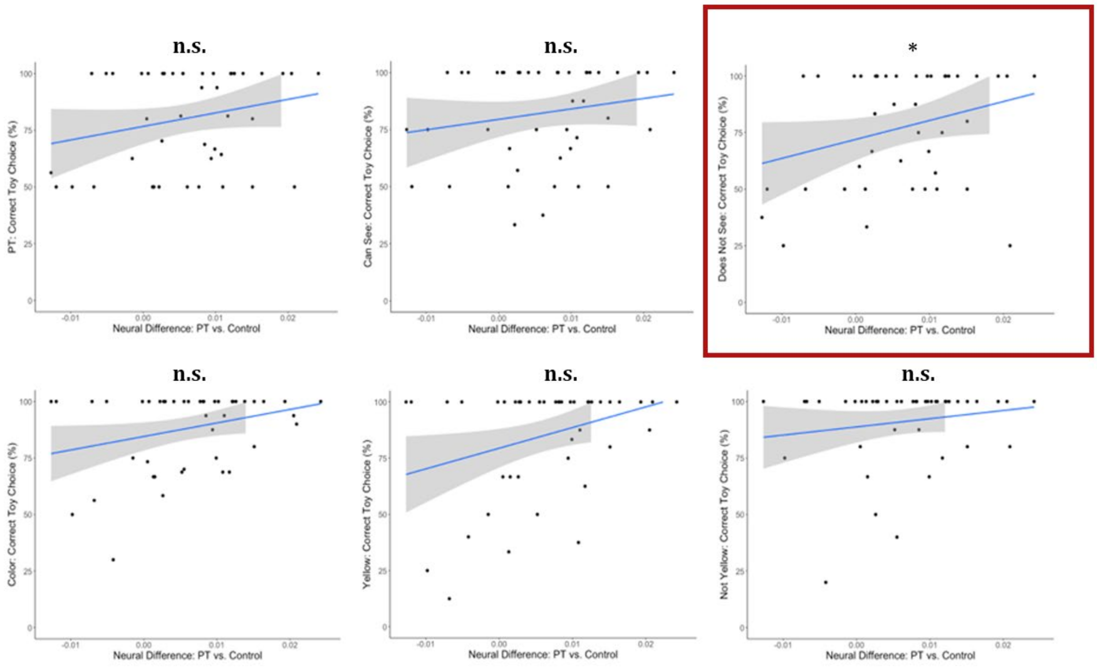

Supplementary Figure 8. Scatter plots illustrating the relation between behavioral performance and neural difference. Dots represent individual participants. Blue line reflects regression line. Shaded area reflects 95% confidence interval. On all graphs, normalized power between perspective-taking and control trials is represented on the x-axis. Top graphs from left to right: Percentage of correct toy choices 1) averaged across all perspective-taking trials, 2) averaged across Can See trials, 3) averaged across Does Not See trials represented on the y-axis. Bottom graph from left to right: Percentage of correct toy choices 1) averaged across all control trials, 2) averaged across Yellow trials, 3) averaged across Not Yellow trials represented on the y-axis.

Additional exploratory analyses.

Age. To investigate for potential effects of children's age on the observed neural effect of our final, post-hoc analysis or children's overt perspective-taking performance, we computed Pearson correlations with children's age in days and the average neural difference between perspective-taking and control trials, as well as the average percentage of correct toy choices on the perspective-taking trials. As a result, we saw no evidence for a correlation between age and the strength of the neural difference ($r(44)$ = -.234, p = .118) or children's perspective-taking performance ($r(44)$ = -.195, p = .194).

TPVT. To explore potential links with language skills, we computed Pearson correlations with children's TPVT scores and the average neural difference, as well as the average percentage of correct toy choices on the perspective-taking trials. The correlation between language skills and perspective-taking performance revealed that the TPVT scores were positively correlated with children's perspective-taking performance ($r(44)$ = .456, p = .001). This is in line with a large body of previous findings on false-belief understanding and language abilities (Milligan, Astington, & Dack, 2007). No evidence for a relation was found when correlating TPVT scores with the neural difference values ($r(44)$ = .080, p = .597).

CSUS. To further explore the potential relation with parental estimations of children's social understanding as administered by the Children's Social Understanding Scale (CSUS) questionnaire (Tahiroglu et al., 2014), we computed Pearson correlations with children's CSUS scores and the average neural difference, as well as the average percentage of correct toy choices on the perspective-taking trials. We found no evidence for a correlation between CSUS scores and perspective-taking performance ($r(44)$ = -.028, p = .853) or CSUS scores and the neural difference values ($r(44)$ = .150, p = .321).

Included vs. excluded participants. The comparison of CSUS scores and TPVT scores between participants who were included versus excluded from the final analysis did not reveal any significant differences. That is, we found no evidence for a difference between CSUS scores (t(58) = -.310, p = .757) between excluded (M = 3.14, SD = .45) and included (M = 3.18, SD = .37) participants. We also

did not find any evidence for a difference between TPVT scores (t(58) = -.935, p = .354) between excluded (M = 99.71, SD = 9.65) and included (M = 102.83, SD = 11.23) participants.

Furthermore, we explored potential differences in children's race/ethnicity and parental education levels for the children included in versus excluded from the final sample. For this purpose, we combined data into binary categories - race/ethnicity: white vs. non-white (included: 27 white, 19 non-white; excluded: 5 white, 9 non-white), education: post-graduate vs. not post-graduate degree (included: 37 post-grad, 9 not post-grad; excluded: 4 post-grad, 10 not post-grad). The results showed no evidence of a significant association between race/ethnicity and inclusion: white vs. non-white ($\chi^2(1) = 1.45$, p = 0.229), though there was a significant association between parental education and inclusion: post-grad vs. not post-grad ($\chi^2(1) = 11.05$, p = 0.001). This suggests that children whose parents had not completed a post-graduate degree may have been more likely to be excluded from the study. The interpretation of these results should be regarded with caution due to the small cell sizes.

Longitudinal participants. Additional analyses were conducted to compare the perspective-taking performance and neural difference between the children who took part in the longitudinal study (i.e., those who participated in the behavioral version of the study at age 3) and those who did not. Results show no evidence for a difference between these two groups for the perspective-taking performance (longitudinal: M = 76.57, SD = 23.45; not-longitudinal: M = 82.32, SD = 20.01; $t(44) = .894$, $p = .376$) nor for the neural difference (longitudinal: M = .0048, SD = .007; not-longitudinal: M =.0061, SD =.01; $t(44) = .492$, $p = .625$). Thus, our data do not speak to any carry-over effects from the longitudinal study.

Trial number. To explore whether the number of included EEG trials was related to the neural effect we observed, we computed Pearson correlations with children's number of perspective-taking and control trials with the average neural difference. We found no evidence for a correlation between the number of trials in the perspective-taking condition and the neural effect ($r(44) = -.028$, $p = .853$) or the number of trials in the control condition and the neural effect ($r(44) = .150$, $p = .321$).

Additional discussion.

While the precise role of temporal-parietal brain areas in perspective-taking, particularly TPj, remains a matter of debate (e.g., Decety & Lamm, 2007; Doricchi et al., 2022; Mars et al., 2012; Schurz et al., 2017), it has been proposed that TPj is involved in self-other distinction (Quesque & Brass, 2019) and the distinction between one's own viewpoint and embodying oneself in the position of another person (Wang et al., 2016). Temporal-parietal areas like posterior TPj are particularly sensitive to the distinction between self and other, which has been reflected in research on inhibition of imitation (Brass et al., 2009). Inhibition of imitation reflects the need to inhibit the automatic tendency to imitate another person so that the other person's actions do not affect one's own performance (Brass et al., 2009; Sowden & Catmur, 2015). Key elements of perspective-taking, namely that one's own perspective may be distinct from another person's viewpoint, requires this self-other distinction. The current neural findings, specifically that the neural effect emerged when the child's visual access differs from their interaction partner's, are in line with these previous findings. In adults, practicing to inhibit the automatic tendency to imitate another person has been shown to facilitate perspective-taking performance (Santiesteban, White, et al., 2012). Interestingly, in a behavioral study with 3-year-old children using the same interactive perspective-taking task as in the current study, we have previously found seemingly contradictory patterns of results (Brezack et al., 2021). That is, children who were more sensitive to another person's actions (i.e., showing an automatic tendency to imitate another person) were better at perspective-taking, specifically when perspectives differed (Brezack et al., 2021). However, in contrast to adults, only about one third of the 3-year-olds showed this automatic tendency to imitate another person at all; thus, the sample was too small to investigate the relation between automatic imitation and perspective-taking. We speculated that children may first need to take their social partner into consideration *at all* (as reflected in their automatic imitation tendencies) before they consider another person's viewpoint. Once children account for their social partner, they might use similar self-other distinction processes as adults when perspective-taking. Indeed, a recent training study by Kampis and colleagues with slightly older children (around 4 years) showed that

practicing inhibition of imitation had a positive effect on children's perspective-taking performance (Kampis et al., 2023). The 4-year-old children, in contrast to the 3-year-olds, successfully incorporated another person's perspective into their behavior even when it differed from their own perspective. In fact, we found the first indication that this differential neural response was positively correlated with children's subsequent perspective-taking performance. It is possible that at age 4, the neural correlate at temporal-parietal sites and its link to perspective-taking behavior may also be associated with the self-other distinction processes that are present when *inhibiting* the automatic tendency to imitate others. This represents a clearly definable and testable hypotheses for future research.

References

Kampis, D., & Southgate, V. (2020). Altercentric cognition: How others influence our cognitive processing. *Trends in Cognitive Sciences*, *24*(11), 945–959.

Milligan, K., Astington, J. W., & Dack, L. A. (2007). Language and theory of mind: Meta-analysis of the relation between language ability and false-belief understanding. *Child development*, *78*(2), 622-646.

Santiesteban, I., Kaur, S., Bird, G., & Catmur, C. (2017). Attentional processes, not implicit mentalizing, mediate performance in a perspective-taking task: Evidence from stimulation of the temporoparietal junction. *NeuroImage*, *155*, 305–311.

Samson, D., Apperly, I. A., Braithwaite, J. J., Andrews, B. J., & Bodley Scott, S. E. (2010). Seeing it their way: evidence for rapid and involuntary computation of what other people see. *Journal of Experimental Psychology: Human Perception and Performance*, *36*(5), 1255.

Surtees, A., Samson, D., & Apperly, I. (2016). Unintentional perspective-taking calculates whether something is seen, but not how it is seen. *Cognition*, *148*, 97–105.

Tahiroglu, D., Moses, L. J., Carlson, S. M., Mahy, C. E., Olofson, E. L., & Sabbagh, M. A. (2014). The Children's Social Understanding Scale: Construction and validation of a parent-report measure

for assessing individual differences in children's theories of mind. *Developmental psychology*, *50*(11), 2485.

Vestner, T., Balsys, E., Over, H., & Cook, R. (2022). The self-consistency effect seen on the Dot Perspective Task is a product of domain-general attention cueing, not automatic perspective taking. *Cognition*, *224*, 105056.
